# I know a dog when I see one: dogs (*Canis familiaris*) recognize dogs from videos

**DOI:** 10.1007/s10071-021-01470-y

**Published:** 2021-03-19

**Authors:** Paolo Mongillo, Carla Eatherington, Miina Lõoke, Lieta Marinelli

**Affiliations:** Laboratory of Applied Ethology, Department of Comparative Biomedicine and Food Science, University of Padua, Viale dell’Università 16, 35020 Legnaro, Italy

**Keywords:** Dogs, Cross-modal, Expectancy violation, Recognition, Species, Videos

## Abstract

Several aspects of dogs’ visual and social cognition have been explored using bi-dimensional representations of other dogs. It remains unclear, however, if dogs do recognize as dogs the stimuli depicted in such representations, especially with regard to videos. To test this, 32 pet dogs took part in a cross-modal violation of expectancy experiment, during which dogs were shown videos of either a dog and that of an unfamiliar animal, paired with either the sound of a dog barking or of an unfamiliar vocalization. While stimuli were being presented, dogs paid higher attention to the exit region of the presentation area, when the visual stimulus represented a dog than when it represented an unfamiliar species. After exposure to the stimuli, dogs’ attention to different parts of the presentation area depended on the specific combination of visual and auditory stimuli. Of relevance, dogs paid less attention to the central part of the presentation area and more to the entrance area after being exposed to the barking and dog video pair, than when either was paired with an unfamiliar stimulus. These results indicate dogs were surprised by the latter pairings, not by the former, and were interested in where the barking and dog pair came from, implying recognition of the two stimuli as belonging to a conspecific. The study represents the first demonstration that dogs can recognize other conspecifics in videos.

## Introduction

Recognition is the ability to identify an item based on previous experience or knowledge and it is crucial for animals to perform appropriate social behaviour towards known others. Recognition is an umbrella term, under which abilities with different degrees of complexity and specificity are grouped: from the univocal identification of individuals, to the relatively simpler classification into meaningful groups (Gherardi et al. 2012). Kin recognition is an example of the latter which attracted substantial attention, having been investigated in a variety of species (see Holmes and Sherman 1983; Mateo 2004), including dogs (Hepper 1994). However, a crucial form of recognition is possibly the ability to recognize individuals as belonging to one’s own species, or conspecific recognition.

A number of studies have looked at different aspects of recognition abilities in dogs. The vast majority looked specifically at visually-based recognition of cues, provided through 2D static stimuli, i.e. photographs. For instance, Adachi and collaborators (2007) showed that dogs looked longer at a picture of their owner’s face when preceded by an incongruent voice, suggesting that dogs had not expected to see their owner. Eatherington and collaborators (2020) provided further evidence of individual human face recognition, by showing that dogs were more likely to approach a picture of their owner’s face compared to that of a stranger’s. Several other studies provide indications about dogs’ recognition of conspecifics, intended here as the ability to identify pictorial representations of dogs as belonging to a group of animals sharing some common features, not to individually recognize other dogs. An early study (Fox 1971) showed that dogs made socially appropriate responses to a life-sized painting of a dog, spending more time sniffing certain regions of the body (e.g. ear, tail or groin). More recently, Range and collaborators (2008) showed that dogs trained to discriminate pictures of dogs could transfer such learning to novel dog pictures; similarly, Autier-Dérian and collaborators (2013) showed that dogs trained to respond to photographs of dogs’ faces could transfer this to other dog faces, regardless of their phenotype, when presented amongst human and other animal faces. Finally, cross-modal paradigms show that dogs appropriately match dog vocalizations and pictorial representations of dogs under various circumstances (Albuquerque et al. 2016; Faragó et al. 2010; Gergely et al. 2019). Collectively, the evidence suggests that dogs may be able to correctly recognise pictorial representations of conspecifics. However, most of the abovementioned studies compared dogs’ responses to conspecific representations to their response towards very different-looking classes of stimuli, including humans or inanimate objects. The lack of comparison with response to representation of more similar stimuli makes the suggestive evidences not conclusive to this regard.

All of the aforementioned studies employed photographs as stimuli. Although methodologically simple, and appropriate to the aims of such studies, a drawback of this approach is that it confines the assessment to dogs’ responses towards static, morphological features of the stimuli being represented. To overcome this limit, animals can be presented with moving visual representations of others (i.e. videos), allowing to incorporate information about motion and, more generally, behaviour. The use of these stimuli is certainly not a novelty in the ethological field. For example, Plimpton and colleagues (1981) showed juvenile bonnet macaques videos of socially diverse behaviours performed by other macaques. They found that the juvenile macaques behaved in a socially appropriate way, acting submissive and seeking contact with their mother when viewing a threatening male, but approaching a passive female. Another demonstration was put forward by Herzog and Hopf (1986) who showed that videos of predators elicited alarm responses by squirrel monkeys, but videos of non-predators did not. The monkeys also reacted to videos of humans as if they were real people, whilst watching them prepare food or when seeing a caretaker who had recently removed a dead neonate and was therefore viewed as a threat. While these are only few examples, the use of videos would have countless applications for the study of dogs’ behaviour, especially in response to social stimuli. Surprisingly, however, research with dogs has not yet seen an extensive use of videos as stimuli. Pongrácz and collaborators (2003) showed that dogs performed above chance in a classical pointing task, where they were shown a projection of an experimenter performing the pointing gesture, implying that dogs perceived the stimulus as a human being. A replica of the same paradigm, in which real-size videos of dogs were projected instead of humans, representing another recent example of the use of videos in dogs’ behavioural research (Balint et al. 2015). Another recent study reported dogs’ differential physiological and behavioural responses to videos of dogs showing asymmetrical tail wagging associated with specific emotional states (Siniscalchi et al. 2013). The dogs’ responses were coherent with such states, suggesting that dogs had recognised the video as representing a dog. Other studies used animated representations of dog motion, in the form of dot displays, not of fully informative videos, to assess dogs’ reactions to the biological motion of conspecifics and of humans (Eatherington et al. 2019; Ishikawa et al. 2018). Relevant to our aim, the study by Eatherington and collaborators (2019) showed that dogs’ looked longer at random dot displays depicting the motion of conspecifics, even when the dots composing the display were randomly rearranged in space, rather than at inverted manipulations of the same stimuli; the same effect was not observed when human stimuli were projected. The finding suggests that dogs are particularly attracted by representations of motion of a quadrupedal animal; however, the lack of control with a non-dog quadrupedal animal species prevents any conclusion about the dogs’ ability to recognise these stimuli at the species level. The same holds true for the previously mentioned paper by Siniscalchi and collaborators (2013).

A first, necessary step towards the use of videos in the study of dogs’ social behaviour is the demonstration that dogs are able to recognize the stimuli being represented. Therefore, the aim of the present study was to assess whether dogs are able to recognize a video representing a dog as a dog. To this aim, we employed a classical cross-modal expectancy violation paradigm, where videos of dogs or of another unfamiliar quadrupedal species were presented after either a dog or another unfamiliar vocalization. According to the expectancy violation paradigm, a non-surprised reaction (i.e. shorter looking time to the area where the stimuli appeared), when matching dog auditory and visual stimuli were presented, than in other conditions, would support dogs’ ability to recognize conspecifics in videos.

## Methods

### Subjects

Thirty-two dogs with their owners were recruited via the database of volunteers at the Laboratory of Applied Ethology in the University of Padua. Seventeen dogs were pure-breeds (one American Staffordshire Terrier, one American Pitbull Terrier, three Australian Shepherds, one Bracco Italiano, three Border Collies, one Boxer Dog, one Bulldog, one Golden Retriever, one Labrador Retriever, one Maremma Sheepdog, one Poodle, one Yorkshire Terrier, one Miniature Pincher) and 15 were mixed-breed dogs (seven small, ≤ 35 cm at the withers; seven medium, > 35 and < 55 cm; one large ≥ 55 cm). The sample consisted of 17 females and 15 males (mean age ± SD: 5.2 ± 3.2 years). Requirements for recruitment were that dogs were in good health, including no apparent sight problems, and at ease in unfamiliar contexts. Also, to ensure that the cow and horse videos, as well as the frog croaking were unfamiliar (see later), dogs with known experience with any of such species were excluded from the study.

### Stimuli

Dogs were exposed to pairs of auditory and visual stimuli, which belonged to either a dog or to another species to which subjects were unfamiliar. The dog vocalization was a recording of a barking bout, composed of two barks. The unfamiliar vocalization was a frog croaking bout, composed of two croaks; such sound was chosen for its similarity with the barking in terms of overall development of the dynamics and noisiness. Both vocalizations had the exact same duration of 0.5 s. The sounds were presented so to produce an average sound pressure of about 58 dB at the site where the dog’s head was, when the sound was played.

The dog video was a black and white recording of a medium sized, mixed breed, light-coated dog walking laterally across a black, rectangular background area. The animal entered the area from one side and walked across it, taking about two and a half complete leg cycles before completely disappearing on the opposite side. The unfamiliar (non-dog) species video was also a black and white recording of either a light-coated cow or horse, walking across the black background area with the same number of strides as the dog video. The size of the animals was reduced to match the size of the dog. Both videos had the same duration (3.0 s), from the first to the last frame in which part of the animal was visible. When projected to the presentation area, the black background area had a height of 150 cm and a width of 190 cm, whereas the animal portrayed in the video had a height of about 75 cm (from ground to the topmost part of the animal) corresponding to the actual, real-life size of the dog portrayed in the video.

### Experimental setting

**Fig. 1:**
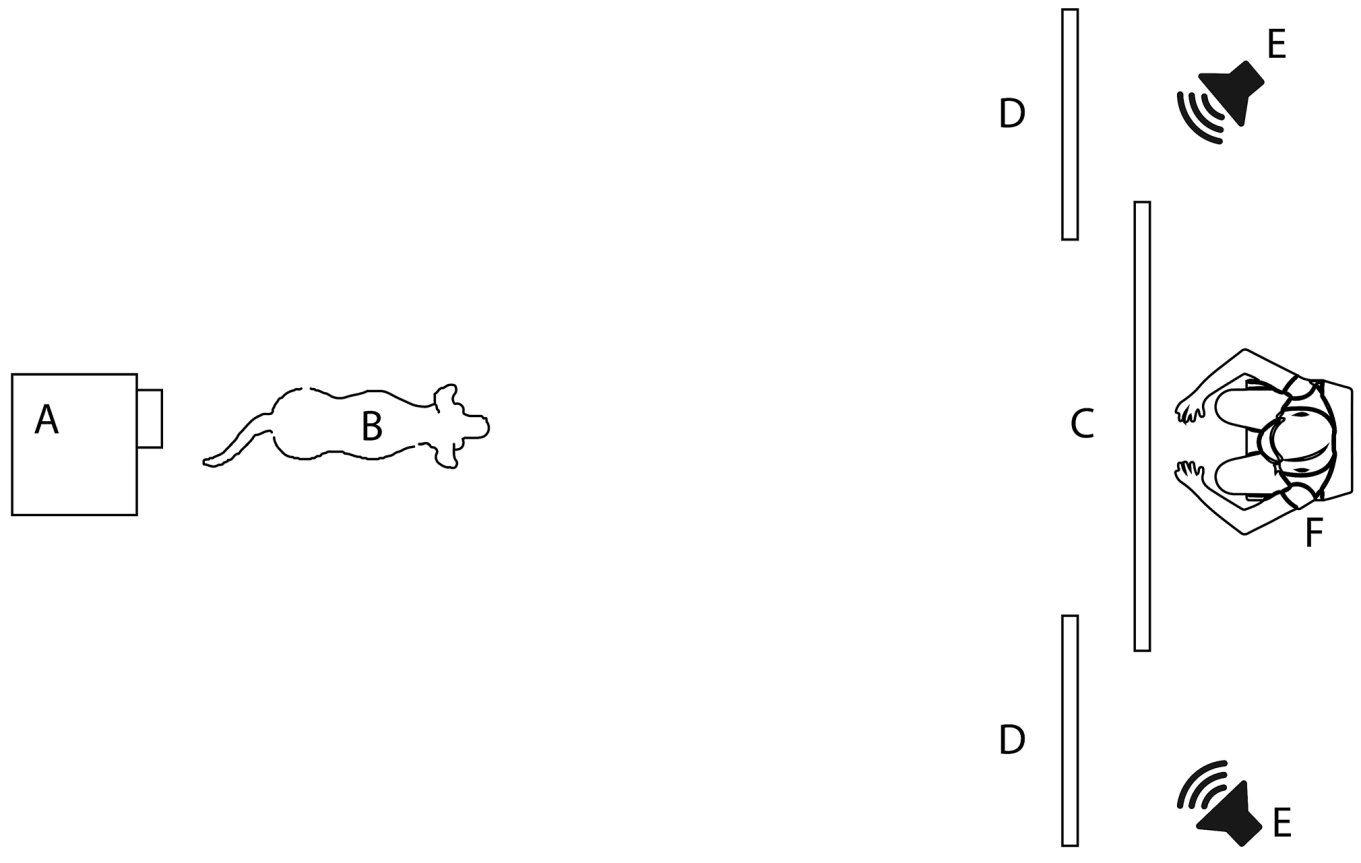
A schematic representation of the experimental setting, illustrating the position of **a** the projector, **b** the dog, **c** the projection screen, **d** the side screens, **e** the speakers and **f** the experimenter operating on the computer during a presentation (figure elements are not to scale)

The experiment was conducted in a quiet, dimly lit room (see scheme in Fig. 1). Along one of the short sides, at approximately 60 cm from it, was a large white plastic screen (150 cm high, and 200 cm wide), which represented the area on which the visual stimuli were projected. Two smaller screens (150 × 100 cm) were placed at the sides of the large one and 10 cm in front of it. During the presentation of stimuli, the side screen created the impression that the animal portrayed in the video appeared from behind a wall. Behind each of the two smaller panels, two active speakers (Hercules XPS 2.0, Hercules Computer Technology, CA, USA) were placed. On the opposite side of the room to the screens was a Toshiba TDP T100 projector mounted 207 cm high on a shelf on the wall. Both the projector and speakers were connected to a MacBook Air laptop (Apple Computers Inc., Cupertino, CA, USA), which was used to control the presentation of the stimuli by an experimenter sitting behind the central panel. During testing, dogs sat or stood at a distance of 240 cm from the screen, and between the legs of their owner who was seated on a small stool behind them. Owners were instructed to gently hold the dog in place and look down at their lap so as not to influence the dog’s behaviour.

Two CCTV cameras mounted on the ceiling captured, respectively, a view of the dog from behind, including the projection area, and a detailed view of the dog from straight above the dog. A Canon XA20 (Canon, Tokyo, Japan) camcorder was mounted over the top of the screen via a tripod and pointed towards the dog’s face; this camera was set in infrared recording mode, allowing for clear detection of the contour of the pupils and eye orientation. The experimenter sitting behind the screen used this camera to see when the dog was looking forward and therefore start the trials.

### Experimental procedure and design of the experiment

At the start of each trial, dogs were led into the room by their owner and positioned, facing the screen at the designated location, marked by tape on the floor. When the dog was in place, the experimenter started the presentation of the stimuli, which entailed the reproduction of the vocalization from one of the two speakers, and the simultaneous reproduction of the video of the animal walking in the projection area from the same side, the vocalization was played from, and disappearing on the opposite side. After the disappearance of the video, the experimenter waited 30 s before eventually turning on the lights. During this interval, the owners were instructed to keep looking at their laps and not to interfere with the behaviour of the dog, except for the gentle restraint. After the 30 s had passed, the experimenter turned on the lights, and owner and dog left the room, waiting for 5 min before entering for the following trial.

All dogs underwent four trials, during which the four possible combinations of dog and non-dog auditory and visual stimuli were presented. The order of presentation of the four combination was balanced within the sample, so that each combination was equally as often presented as the 1st, 2nd, 3rd and 4th trial. For half of the dogs in the sample, the visual stimulus was represented by the horse, and for another half by the cow.

### Data collection and analysis

Using Observer XT software (version 12.5, Noldus, Groeningen, The Netherlands), a continuous sampling technique was used to collect data about dogs’ orientation, which was coded as either looking centrally (towards the central part screen area), looking at the entrance side or looking at the exit side (respectively, the side of the presentation area where the projected animal came in from or left at). Data were collected in an interval of time spanning the frame when a part of the animal was first visible, until 30 s after the animal had disappeared from the screen.

For the aim of analysis, collected data were split in two different time intervals: one relative to when the projected animal was visible, one relative to the 30 s following its disappearance. For each interval, a set of four variables were obtained: the total time spent looking at the entrance, centrally, or at the exit, and at the entire presentation area (the latter representing the sum of the first three variables). The rationale for dividing data collection into two intervals, was that we expected dogs’ attention to be primarily driven by the presence of the stimuli while the latter were projected; conversely, after the stimuli had disappeared, dogs’ attention would be more indicative of possible surprised reactions to expectations induced by the pairing of stimuli. To assess whether dogs’ attention was indeed driven by the presence of the stimuli when these were projected, data collected in such interval were further split into three equally long sub-intervals (1 s) corresponding to the stimulus occupying the entrance, central and exit region of the presentation area, respectively.

Inter-observer reliability for dogs’ head orientation data was assessed using data collected by a second observer on a randomly selected subset of videos (*N* = 18, ~ 30% of the total number); a Pearson’s correlation coefficient of 0.89 was obtained between data collected by the two observers, supporting the reliability of data collection.

Data analysis was based on Generalised Estimating Equation (GEE) models. A first model was run to assess whether dogs’ overall attention to the presentation area remained stable across the four presentations or any decrement in attention was observed. The model included the dogs’ name as a random factor accounting for repeated measures taken from each dog, and the order of trials (1–4) as a fixed factor. The dependent variable was the total attention to the presentation area. Corrected post-hoc comparisons were run to assess pairwise differences between trials presented at a different place in the sequence. Models were run separately for data collected when the projected animal was present, and after its disappearance.

The next analysis assessed whether the type of visual or auditory stimulus, or their combination, had an effect on dogs’ orientation, either during stimulus presentation or after the stimulus had disappeared. GEE models were run with the dogs’ name as random factor to account for repeated measurement within each dog. The model included as fixed factors the type of visual stimulus (dog, non-dog), the type of auditory stimulus (bark, croak), and their interaction; to assess potential differences between the horse and cow video, the model also included the effect of the type of non-familiar species (cow, horse), as a nested factor within the type of visual stimulus and in interaction with the type of auditory stimulus. Furthermore, to assess whether dogs’ allocation of attention to different parts of the presentation area was driven by the movement of the stimulus, the location of the stimulus (at entrance, central, or exit region) was also included as a fixed factor. Different models were run, using as dependent variables, the time spent looking centrally, at the entrance or at the exit side, respectively, while the stimulus was present and after its disappearance. Corrected post-hoc comparisons were run to assess pairwise differences between trials presented at a different place in the sequence.

Analysis was performed with SPSS (ver. 26; IMB, Armonk, NY). Results are reported as mean ± SD unless otherwise stated.

## Results

During the presentation of the stimuli, dogs spent on average 2.8 ± 0.4 s (min: 0.6, max: 3.0) oriented to the presentation area, with no significant difference between trials presented in different order (Wald Chi-square = 4.3, *P* = 0.23, GEE). However, the order of trial presentation had an effect on the length of time dogs were oriented to the presentation area after the stimulus had disappeared (Wald Chi-square = 17.3, *P* = 0.001); specifically, no difference was found between the 1st (estimated mean ± SD = 20.0 ± 1.5 s) and the 2nd trial (20.2 ± 1.5 s), but the time spent looking at presentation area decreased significantly in the 3rd (17.7 ± 1.7 s; *P* = 0.026) and the 4th trial (15.5 ± 1.5 s; *P* < 0.001). To adopt a conservative approach, we therefore decided to limit further analysis of dogs’ orientation after the disappearance of the stimuli to data of the 1st and 2nd trials. Conversely, data from all four trials were analyzed for dogs’ orientation while stimuli were projected.

**Fig. 2:**
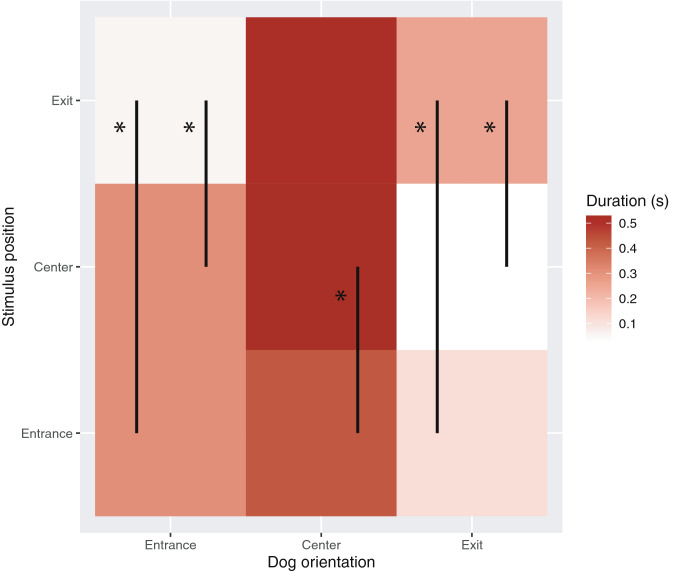
Heat-map representing the percentage of time spent by dogs’ oriented to different region of the presentation area, as a function of the region predominantly occupied by the projected visual stimulus. Vertical lines indicate significant differences in means (*P* < 0.05, Bonferroni-corrected post-hoc comparisons after Generalized Linear Equation Models)

**Fig. 3:**
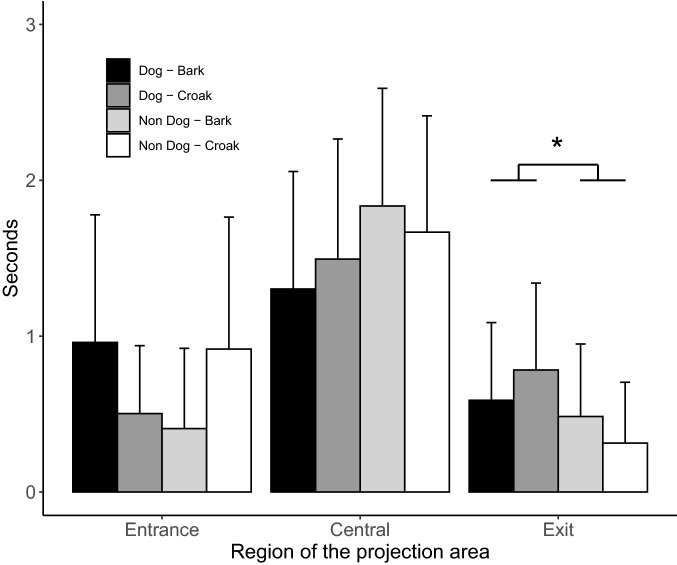
Mean ± SD time (s) spent looking at the different regions of the presentation area while any part of the stimulus was visible on it, as a function of the stimuli pair (*P < 0.05, Bonferroni-corrected post-hoc comparisons after Generalized Linear Equation Models)

During the presentation of the stimuli, dogs spent an average of 1.6 ± 0.8 s looking centrally, 0.7 ± 0.7 s looking at the stimulus entrance side, and 0.5 ± 0.5 s at the stimulus exit side. Table 1 summarizes the results of the GEE indicating the effects of the type of stimuli presented and of the region occupied by the stimulus on the projection area on the dogs’ orientation variables during the presentation of the stimuli. The region where the stimulus was projected significantly affected all orientation variables, as shown through the heatmap in Fig. 2. In regard to the type of stimuli presented, the time spent oriented centrally was not affected by either the visual or auditory stimulus. The time spent looking at the entrance side was affected by an interaction between the two factors: however, after applying corrections for multiple comparisons, no significant difference was found between different levels of the interaction. The time spent looking at the exit was affected by the type of visual stimulus, with longer looking observed when a video of a dog was presented, than when a non-dog video was presented (Fig. 3).

**Table 1:** Generalized Estimating Equations model assessing the effects of the type of visual stimulus (dog/non-dog), the species of the non-dog and of auditory stimulus on time spent by dogs looking centrally, at the entrance side or at the exit side, during and after the presentation of the stimuli

Factor	Looking at entrance side	Looking centrally	Looking at exit side
Region occupied by stimulus	*Χ*^2^ = 90.04 *P* < 0.001	*Χ*^2^ = 8.69 *P* = 0.013	*Χ*^2^ = 31.44 *P* < 0.001
Visual stimulus	*Χ*^2^ = 0.04 *P* = 0.843	*Χ*^2^ = 3.10 *P* = 0.078	*Χ*^2^ = 7.98 *P* = 0.005
Auditory stimulus	*Χ*^2^ = 1.74 *P* = 0.187	*Χ*^2^ = 1.44 *P* = 0.231	*Χ*^2^ = 0.017 *P* = 0.896
Auditory stimulus × Visual stimulus	*Χ*^2^ = 5.71 *P* = 0.017	*Χ*^2^ = 2.50 *P* = 0.114	*Χ*^2^ = 1.19 *P* = 0.290
Auditory stimulus × Species of non-dog visual stimulus (nested within visual stimulus)	*Χ*^2^ = 1.34 *P* = 0.501	*Χ*^2^ = 4.77 *P* = 0.098	*Χ*^2^ = 1.51 *P* = 0.468

**Fig. 4:**
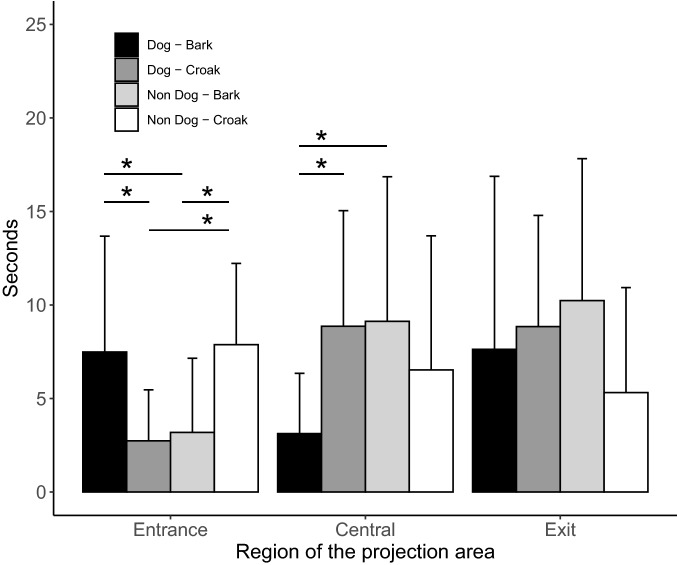
Mean ± SD time (s) spent looking at the different regions of the presentation area after the stimuli had disappeared, as a function of the stimuli pair (*P < 0.05, Bonferroni-corrected post-hoc comparisons after Generalized Linear Equation Models)

Table 2 summarizes the results of the GEE indicating the effects of the type of visual stimulus, vocalization, and their interaction on dog’s orientation variables during the presentation of the stimuli. After the stimulus had disappeared, dogs looked centrally for a mean ± SD of 6.9 ± 6.6 s, at the entrance side for 5.3 ± 4.9 s and at the exit side for 8.0 ± 7.3 s. The time spent looking centrally was affected by an interaction between the type of visual and the type of auditory stimulus, with shorter time spent when a video of a dog was paired with barking, than when either of the dog stimuli was paired with a non-dog counterpart (P < 0.05). The pairing of non-dog stimuli resulted in intermediate amounts of attention, not different from any other stimulus combination (Fig. 4). The interaction between visual and auditory stimulus also affected the time spent looking at the entrance side, which was longer in the case of matching pairs (dog + barking, or non-dog + croaking), than when the auditory and visual stimuli did not match (*P* < 0.05) (Fig. 3). No effect of the type of visual or of auditory stimulus was found for the time spent looking at the exit.

**Table 2:** Generalized Estimating Equations model assessing the effects of the type of visual and of auditory stimulus on time spent by dogs looking centrally, at the entrance side or at the exit side, after the presentation of the stimuli

Factor	Looking at entrance side	Looking centrally	Looking at exit side
Visual stimulus	*Χ*^2^ = 0.14 *P* = 0.708	*Χ*^2^ = 1.90 *P* = 0.168	*Χ*^2^ = 0.09 *P* = 0.768
Auditory stimulus	*Χ*^2^ = 0.00 *P* = 0.985	*Χ*^2^ = 0.85 *P* = 0.355	*Χ*^2^ = 2.00 *P* = 0.157
Visual stimulus × auditory stimulus	*Χ*^2^ = 19.13 *P* < 0.001	*Χ*^2^ = 12.09 *P* = 0.001	*Χ*^2^ = 2.30 *P* = 0.129
Auditory stimulus ×Species of non-dog visual stimulus (nested within visual stimulus)	*Χ*^2^ = 1.18 *P* = 0.880	*Χ*^2^ = 4.553 *P* = 0.336	*Χ*^2^ = 5.91 *P* = 0.206

## Discussion

In this study, we employed a cross-modal, expectancy violation paradigm to assess whether dogs can recognize the species of conspecifics from videos. Dogs were presented with pairs of auditory and visual stimuli, which could be any combination of dog-related on non-dog-related vocalization and video. Dogs’ orientation towards the presentation area, as a function of the presented pair of stimuli, was analysed during two time intervals, in which different mechanisms were most likely at play.

The first interval spanned from the onset of the vocalization to the last frame in which the video of the animal crossing the screen was visible. Dogs’ orientation in this interval therefore reflected a proximate reaction to the presence of the stimuli, rather than an after-effect of the pairing.

Dogs spent almost the entire interval oriented toward the projection area. Moreover, dogs’ attention to specific regions of the projection area roughly followed the stimulus occupation of such regions. This finding is most likely a direct result of the capacity of motion stimuli to elicit orientation responses, an effect that is particularly relevant for stimuli abruptly appearing within the visual field (Hillstrom and Yantis 1994) and for stimuli depicting animate entities (Pratt et al. 2010), two features that characterised the visual stimuli that were presented in this experiment.

A breakdown analysis of dogs’ orientation to the different parts of the projection area revealed that dogs spent longer time looking at the exit area when a dog video was projected than when the unfamiliar species was projected. Therefore, dogs were more likely to visually follow the dogs’ video until it left the presentation area, than the unfamiliar species video. The finding is consistent with the notion that familiarity drives attentional responses for visual stimuli (Christie and Klein 1995). There is some direct evidence that this process also applies to dogs, in particular when presented with representations of dogs’, such as face photographs (Racca et al. 2010) or biological movement (Eatherington et al. 2019). Overall, the findings support the idea that dogs did at least perceive the dog video as a familiar stimulus.

Evidence that dogs did recognise the dog-related stimuli as belonging to a dog, however, comes from the analysis of attention patterns after the stimuli had disappeared. In this time interval, dogs spent less time oriented towards the central part of the presentation area when a bark was followed by the appearance of a dog video, than when any of such two stimuli was paired with an unfamiliar counterpart. In accordance with the violation of expectancy paradigm, longer looking at the main projection area reflected a surprised reaction to the pairing of an unfamiliar-species stimulus with a dog stimulus. Analogous interpretations of longer looking times have been found in studies in dogs (Adachi et al. 2007) and other species including cats (Takagi et al. 2019), horses (Lampe and Andre 2012; Nakamura et al. 2018), crows (Kondo et al. 2012) and lions (Gilfillan et al. 2016). Therefore, this result clearly indicates that dogs perceived the appearance of the dog video as an expected consequence of the barking, implying they had appropriately recognized both stimuli as belonging to a dog. Following presentation of dog stimuli, dogs also spent longer time looking at the entrance region of the presentation area, than when either dog stimulus was paired with an unfamiliar-species stimulus. No such effect was observed for attention to the exit region. Although the reason for this pattern of results is not immediately clear, we believe the result is further indication that dogs retained the pair of dog stimuli as coherently representing a dog; in this sense, dogs may have been interested in where the animal came from, especially since nothing indicated the presence of such animal before its sudden appearance. The lack of differences in attention to the exit region, on the other hand, could reflect a relatively low need to monitor an animal who was moving away from the observer.

When both stimuli belonged to an unfamiliar species, the pattern of dogs’ attention to the presentation area was less clear-cut than those observed when presented with dog stimuli. On the one hand, attention to the central part of the presentation area when non-dog stimuli were paired was not different than that observed when dog stimuli were paired. The similarity in reaction may suggest dogs considered the appearance of the unfamiliar individual as a plausible consequence of the unfamiliar vocalization, much as they considered the appearance of the dog an unsurprising consequence of the bark. Unsurprised reactions to pairs of unfamiliar stimuli in an expectancy violation test have also been reported before (e.g. Adachi et al. 2007). As already discussed for the pair of dog stimuli, the high amount of attention paid to the entrance region could indicate the interest in where an unknown (but plausible) type of animal came from. On the other hand, dogs’ attention to the central part of the presentation area after non-dog stimuli pairs were presented was also not lower than when a dog/non-dog stimuli pair was presented. A possible explanation is that dogs’ attention patterns after being exposed to the two unfamiliar stimuli was driven by the interest in such novel stimuli, rather than by a violated expectation. Indeed, different studies showed neophilic reactions by dogs (e.g. Kaulfuß and Mills 2008; Racca et al. 2010). Of particular relevance, as it deals with visual preference, the study by Racca and collaborators (2010) showed that while dogs pay preferential attention to familiar rather than novel images of dogs, the opposite is true for other classes of stimuli, including images of objects or of human faces. Along this reasoning, hearing a novel auditory stimulus drove attention to the entrance region, and seeing a novel visual stimulus drove attention to both the entrance and central region (the latter being predominantly occupied when the stimulus became fully visible).

One question arising from our results whether dogs showed a different response to the pairing of the bark and dog video merely because they were familiar with both stimuli, without implying classification of the stimuli as belonging to a dog. The literature provides some indications that this may not be the case. For instance, Gergely and collaborators (2019) showed that dogs exposed to a conspecific vocalization pay more attention to pictures of dogs than of humans, a species dogs were highly familiar with. Moreover, a recent functional neuroimaging study revealed greater activation of visual cortical areas in dogs, when exposed to videos of conspecific faces than when exposed to human faces, suggesting the existence of species-specific processing mechanisms (Bunford et al. 2020). Taken together, these findings suggest dogs do possess the ability to visually discriminate dogs from another familiar species. Whether such ability is the result of exposure alone or is aided by a predisposition is impossible to state by the results of the present or of other studies in dogs. Findings in humans indicate that experience builds on top of predispositions in determining one’s ability to identify motion features as belonging to a conspecific (reviewed by Hirai and Senju 2020). A thorough understanding of if and how the same factors impact on dogs’ ability to recognize other animals would require further experiments, which are currently ongoing in our laboratory.

Few other studies have attempted to demonstrate dogs’ ability to recognize the species of other conspecifics in figurative representations, providing suggestive though not conclusive evidence (Autier-Dérian et al. 2013; Gergely et al. 2019). The present findings differ in important ways from all previous attempts. First, in all other studies, the stimuli depicted animal heads, whereas our stimuli represented lateral views of the animal’s whole body. Our findings imply that a detailed frontal view of the head is not a necessary stimulus for dogs to recognize a conspecific, at least if motion information is available. Indeed, a crucial difference between the present and earlier studies was that we presented videos rather than still images, allowing us to incorporate information about movement. Our own laboratory showed dogs are attracted by the motion of a laterally walking dog (Eatherington et al. 2019) and studies in other species highlight how motion cues alone can be used for the recognition of conspecifics (Jitsumori et al. 1999; Nunes et al. 2020). Thus, the presence of motion information in our experiment may have played a role in allowing dogs to appropriately identify the conspecific’s video. The abovementioned studies indicate that morphology, independently from motion, can also be individually sufficient to the aims of recognition (Jitsumori et al. 1999; Nunes et al. 2020). However, these studies only depicted heads, a stimulus that is rich in features useful to the aims of recognition, even to the level of the individual. Our findings indicate that even more limited morphological details provided by a lateral, whole body view, paired with motion information may be sufficient for dogs to recognize a conspecific.

Finally, research on dog visual cognition has used the cross-modal and expectancy violation paradigms; for instance, similar paradigms have been successfully used to demonstrate dogs’ recognition of humans’ identity or sex (Adachi et al. 2007; Ratcliffe et al. 2014), or expectations about conspecifics’ body size (Taylor et al. 2011). However, to the best of our knowledge, this method had never been used in dogs with videos and some methodological considerations seem useful at this stage. First, while videos were projected, dogs spent most of their time oriented towards the presentation area, indicating the stimuli were able to attract the dogs’ attention (at least from a behavioural standpoint), a crucial and often problematic aspect of research on visual cognition. Second, even after the stimulus disappeared, dogs remained oriented towards the presentation area for a significant portion of the allowed 30 s—suggesting maintenance of interest in what had been projected. Third, the analysis of dogs’ orientation across subsequent presentations suggests limited habituation through the first two trials, but a significant decrement starting from the third trial. Overall, these results indicate the method is suitable to study dogs’ spontaneous cross-modal processing of auditory and animated visual stimuli, and that dogs can be presented with up to two presentations before their attention starts to decline.

## Conclusion

This study provides the first evidence that dogs recognize videos of dogs as actually representing dogs. These findings will hopefully be a starting point towards the more extensive use of videos in dog behavioural and cognitive research. At the same time, several questions arise from our results; for instance, our stimuli depicted a laterally walking dog, but it would be important to assess whether recognition extends to other dynamic behaviours. A related question is whether motion information alone would be sufficient for dogs to recognize dogs in videos or if, in fact, other figurative information (e.g. shape, color, etc.) is needed for recognition. Finally, as some of our findings suggest a role of experience or familiarity with the class of stimuli, more studies are needed to determine how exposure impacts the dogs’ ability to recognize conspecifics or other species in videos.

## Data Availability

Data are publicly available in the data repository of the University of Padua.
